# Supplementary figures and images for: Neuronal microscale biophysical instability mediates macroscale network dynamics shaping pathological manifestations
Source: bioRxiv. 2026 Apr 4:2026.01.20.697254. Originally published 2026 Jan 22. Preprint. [Version 2] doi: 10.64898/2026.01.20.697254 (PMC12871810; doi:10.64898/2026.01.20.697254)

A

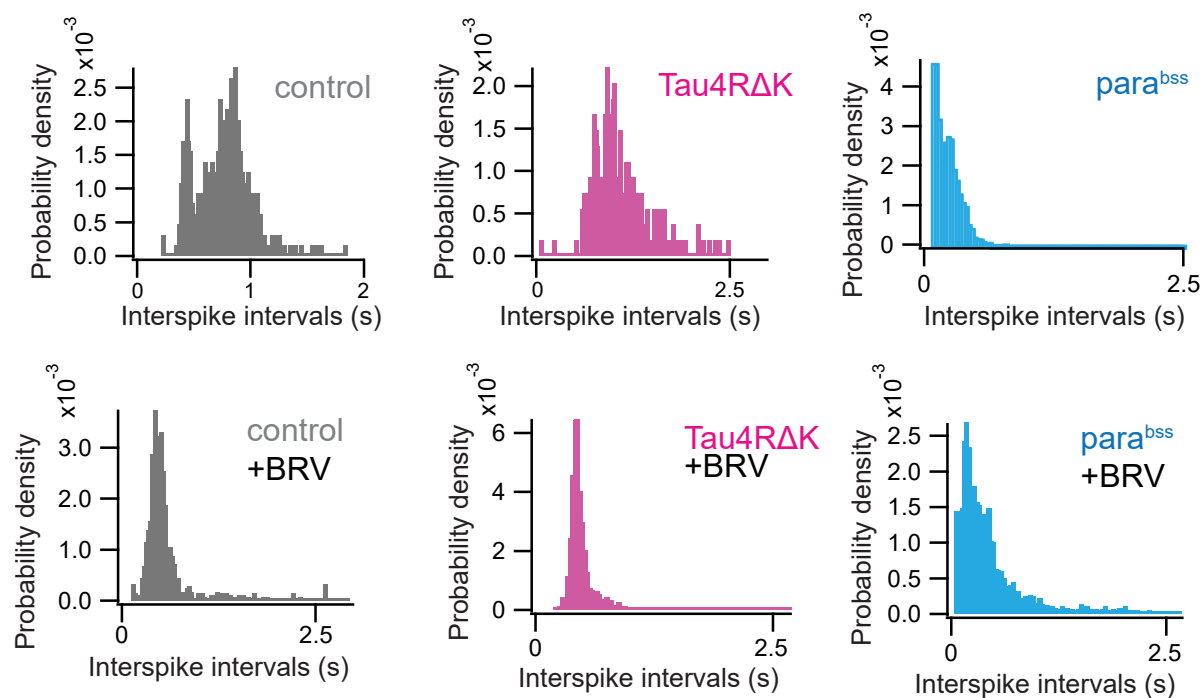

B

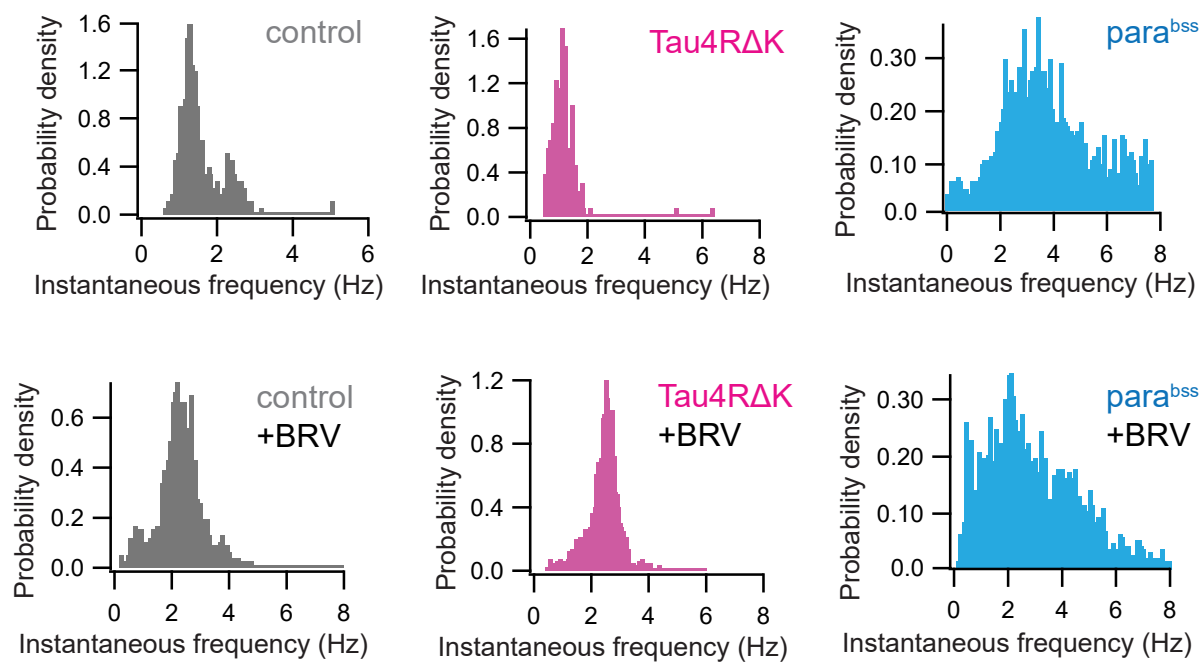

Fig S1

A

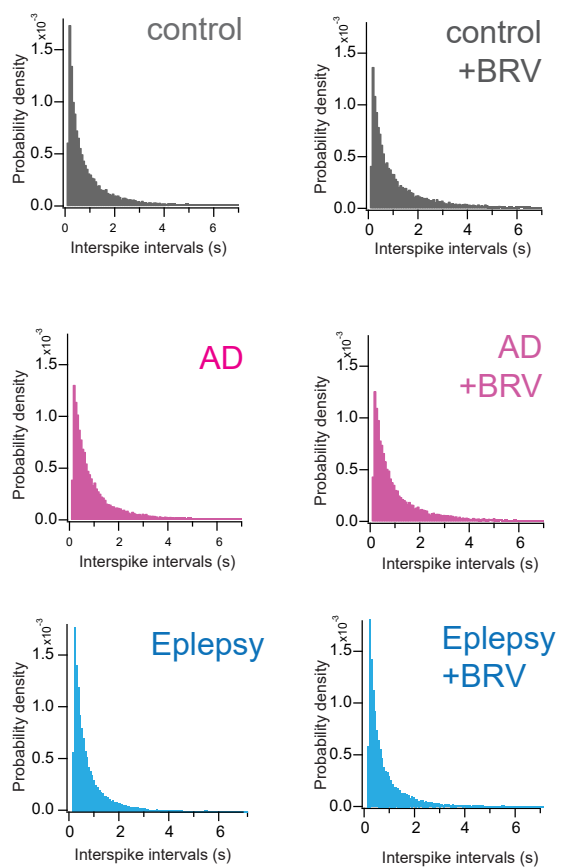

B

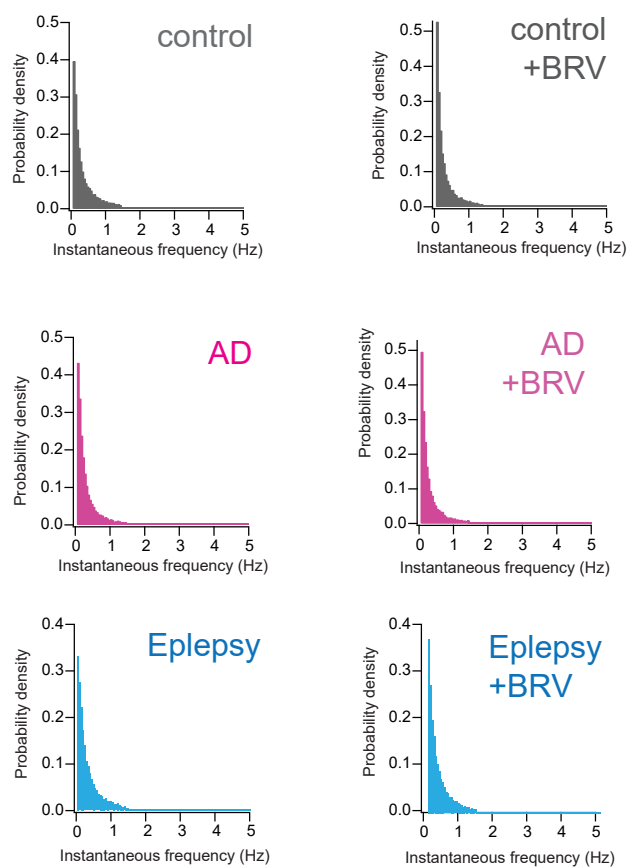

Fig S2

Supplement: Supplement 1 — Fig S1: Additional electrophysiological analysis for the data used in Figs.1 and 2. (A) Probabilistic distribution structure of interspike intervals distribution and (B) probabilistic distribution structure of instantaneous spike frequency obtained from spontaneous firing in DN1p circadian neurons with/without Tau4RΔK or parabss expression. Fig S2: Additional electrophysiological analysis for the data used in Fig.4. (A) Probabilistic distribution structure of interspike intervals distribution and (B) probabilistic distribution structure of instantaneous spike frequency obtained from spontaneous firing in human iPSC-derived neuronal culture obtained from AD and epilepsy patients. [file media-1.pdf]
